# A New Method for the Determination of Total Content of Vitamin C, Ascorbic and Dehydroascorbic Acid, in Food Products with the Voltammetric Technique with the Use of Tris(2-carboxyethyl)phosphine as a Reducing Reagent

**DOI:** 10.3390/molecules28020812

**Published:** 2023-01-13

**Authors:** Artur Mazurek, Marzena Włodarczyk-Stasiak

**Affiliations:** Department of Analysis and Evaluation of Food Quality, Faculty of Food Science and Biotechnology, University of Life Sciences in Lublin, Skromna Street 8, 20-704 Lublin, Poland

**Keywords:** vitamin C, ascorbic acid, dehydroascorbic acid, tris(2-carboxyethyl)phosphine, voltammetry, chromatography, validation

## Abstract

The objective of the study was to develop a new method for the determination of the total content of vitamin C and dehydroascorbic acid in food, based on the technique of differential pulse voltammetry with the use of a boron-doped diamond electrode modified with mercury film. A comparison was made between the results obtained with the developed method and a proposed reference method based on high-performance liquid chromatography with spectrophotometric detection. The reduction of dehydroascorbic acid was performed with the use of tris(2-carboxyethyl)phosphine. The interference caused by the presence of tris(2-carboxyethyl)phosphine during the voltammetric determination of ascorbic acid was effectively eliminated through a reaction with N-ethylmaleimide. The conducted validation of the voltammetric method indicated that correct results of analysis of the total content of vitamin C and ascorbic acid were obtained. Analysis of the content of dehydroascorbic acid was imprecise due to the application of the differential method. The results of the analyses and the determined validation parameters of the developed method are characterised by a high degree of conformance with the results obtained with the chromatographic reference method, which indicates the equivalence of the two methods.

## 1. Introduction

The name vitamin C covers ascorbic acid (AA) and dehydroascorbic acid (DHAA), as both forms display high biological activity. Dehydroascorbic acid displays the properties of vitamin C, as it is easily reduced in the organism to ascorbic acid. One of the most important biological functions of ascorbic acid, resulting from its reducing properties, is its capacity for stopping the radical chain reaction [1]. In addition, vitamin C performs several diverse functions in the human organism. The best-researched role of the acid in the human organism is its participation in the synthesis of collagen [2]. The importance of vitamin C in keeping the human organism in good health has been extensively documented. Human beings cannot produce it on its own, as it does not possess the required enzymes, similar to numerous animal species, e.g., Osteichthyes, commonly called bony fish, Passeridae (sparrow family), bats, and domestic guinea pigs [3]. Therefore, the only source of vitamin C for humans is the diet, and approximately 90% of the entire intake of this vitamin comes from plant food products [4]. Ascorbic acid and dehydroascorbic acid occur in all plants, but their content varies extensively among the various plants and species [5]. The stability of vitamin C in food depends on numerous factors, such as, e.g., the composition of the product, oxygen content, pH value, and the methods of processing. All of them have to be taken into account to minimize changes in the content of vitamin C during the life of the food product. However, even if all the conditions are maintained at optimum levels, its content decreases in the course of food processing and storage, and an increase is observed in the percentage of dehydroascorbic acid in the total content of vitamin C in the product [6,7].

In food analysis, the correct method for the determination of vitamin C should measure the combined content of ascorbic acid and dehydroascorbic acid to determine the total content of vitamin C. There are a large number of methods permitting direct analysis of ascorbic acid based on its physicochemical properties, as opposed to dehydroascorbic acid [8,9,10]. For this reason, many of the methods referred to as methods for the determination of vitamin C, in reality, allow only the determination of the content of ascorbic acid, which leads to erroneous results. In relation to the small number of analytical methods making use of direct measurement of dehydroascorbic acid, the most frequently applied procedure is the use of its reduction, leading to the formation of ascorbic acid. To determine the total content of vitamin C, one should add a reducing reagent to the sample, and then determine the content of ascorbic acid, which will be the sum of its initial content and that which appeared as a result of the reduction. To determine the content of dehydroascorbic acid, it is necessary to perform an additional assay of the content of ascorbic acid in the sample, without the reduction stage, and then deduct the obtained result from the total content of vitamin C (subtraction method) [11]. 

High-performance liquid chromatography (HPLC) is undoubtedly the most extensively used technique for determining vitamin C in food products. The advantages of this method include high specificity, sensitivity, and relative ease of performing the analysis. Ascorbic acid is electrochemically active, which allows for the development of numerous analytical procedures, making use of polarographic and voltammetric techniques. Procedures involving the use of the dropping mercury electrode are less frequently employed, giving ground to the voltammetric technique, which makes use of electrodes made of various materials. In many cases, the surface of the electrodes is modified to improve their analytical properties [12,13,14,15,16]. Boron-doped diamond is a modern electrode material which is characterized by excellent properties: wide potential window in water solutions, low background current, long-lasting response stability, and low sensitivity to dissolved oxygen [17]. An unmodified boron-doped diamond electrode (BDE) was used for the development of a procedure for the determination of ascorbic acid in pharmaceutical products and human urine [18,19,20]. A boron-doped diamond electrode modified through the application of a film of lead and Nafion on its surface was used for the determination of ascorbic acid and paracetamol [21]. The primary shortcoming of the polarographic and voltammetric methods used for the analysis of vitamin C is the lack of any possibility of direct determination of dehydroascorbic acid due to the absence of electrochemical activity. It is, therefore, necessary to apply the stage of reduction of that acid to ascorbic acid before the analysis proper can be performed. According to our knowledge, such an approach to the analysis of vitamin C in food was applied only by Lento et al. [22] and in our previous publication [23], in which we described a method of determination of the total content of vitamin C and dehydroascorbic acid in food with the technique of differential pulsed polarography using dithiothreitol (DTT) as the reducing reagent. 

Tris(2-carboxyethyl)phosphine (TCEP) is a compound with an application similar to that of dithiothreitol, which can be considered as an alternative. As opposed to dithiothreitol, it reacts within a broad range of pH values and does not undergo oxidation by atmospheric oxygen, and does not have the unpleasant smell characteristic of thiols [24]. Tris(2-carboxyethyl)phosphine was first used in the analysis of vitamin C in 2000 by Lykkesfeldt [11]. Since then, its importance in the analysis of vitamin C has been growing, due to the possibility of dehydroascorbic acid reduction in an acidic environment. The reduction of dehydroascorbic acid with the use of dithiothreitol requires the stage of elevation of the pH value of vitamin C extract; that stage can be left out when using TCEP, which greatly simplifies the analysis. In addition, very often the voltammetric methods developed for the analysis of ascorbic acid are used only for the analysis of samples with relatively simple matrices, e.g., pharmaceutical, and food is only analyzed in the form of juice samples [12]. Therefore, there is a need for a voltammetric method that will allow the determination of the total content of vitamin C and dehydroascorbic acid in samples of diverse foods. 

In view of the above facts, the objective of the study presented herein was to develop a new voltammetric method for the determination of the total content of vitamin C and dehydroascorbic acid in food with the use of a boron-doped diamond electrode. It was assumed that the derivatization of dehydroascorbic acid will be conducted with the use of tris(2-carboxyethyl)phosphine (TCEP) which, at present, is the most frequently used reagent capable of DHAA reduction in extraction environments with pH < 2. A comparison was made between the validation parameters of the developed voltammetric method and a reference method based on the use of high-performance liquid chromatography with diode-array detection (HPLC-DAD).

## 2. Results and Discussion

### 2.1. Method Development

**Figure 1:**
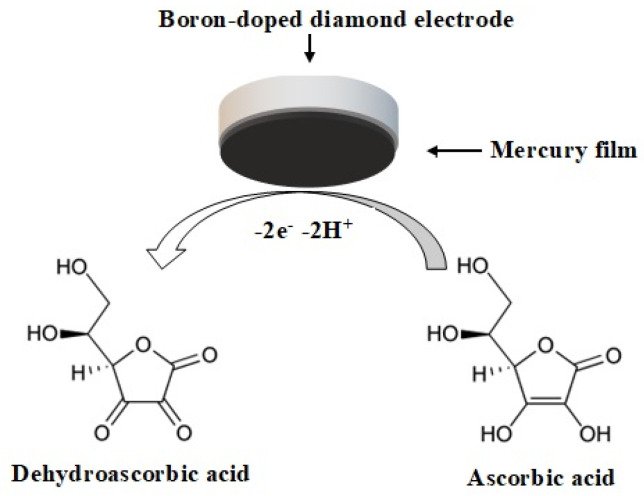
Electrochemical mechanism of ascorbic acid oxidation at the boron-doped diamond electrode modified with a mercury film.

At the initial stage of the research, a voltammetric analysis of ascorbic acid in food samples (orange juice, parsley, kiwi) was carried out on a non-modified boron-doped diamond electrode, using the parameters described in the publication by Sadok et al. [18]. Interference from the matric components of the analysed samples was noted, and manifested as distortion of the voltammogram lines that made it impossible to obtain correct results of the analysis. This made it necessary to modify the surface of the BDE to allow the determination of ascorbic acid in diverse food samples. It was decided to apply a modification consisting in electrochemical application of a film of mercury on the surface of the BDE. The choice of mercury was determined by the use of this element in polarographic determinations of ascorbic acid in numerous and varied food samples, the results of which did not differ statistically significantly from results obtained with the use of the method of high-performance liquid chromatography [25]. The application of mercury film on the surface of the BDE helped to minimise the possibility of the occurrence of interference during the determination of vitamin C in food samples. The mechanism of the electrochemical reaction of ascorbic acid on the mercury electrode described by Ono et al. [26] and Ruiz et al. [27] is schematically shown in Figure 1.

**Figure 2:**
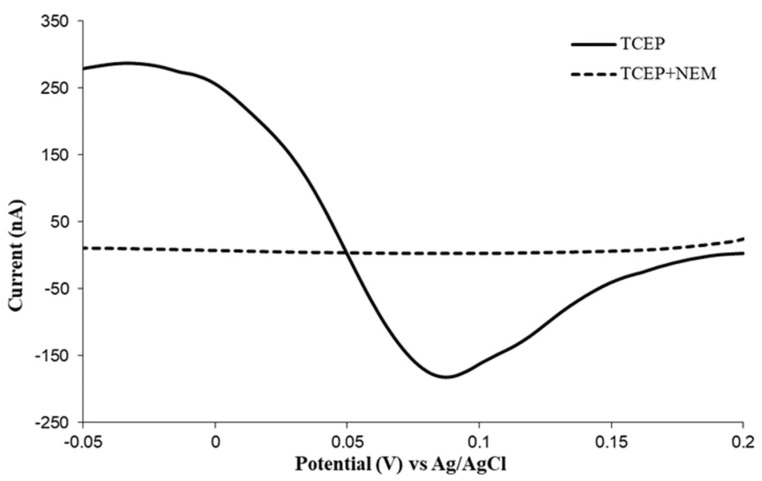
Voltammogram of tris(2−carboxyethyl)phosphine solutions (TCEP) and tris(2−carboxyethyl)phosphine with addition of N−ethylmaleimide (TCEP + NEM).

**Figure 3:**
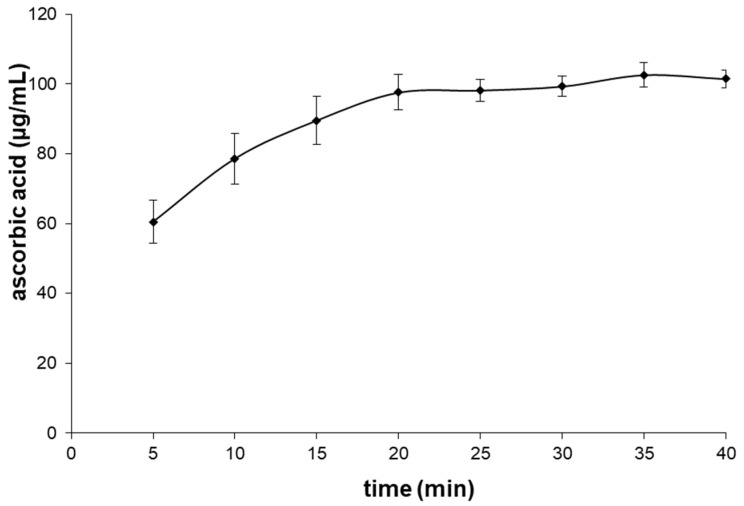
The concentration of ascorbic acid solution (100 µg/mL) determined with the voltammetric technique in the presence of tris(2−carboxyethyl)phosphine versus the time of reaction with N−ethylmaleimide.

At the next stage of the study, it was tested whether tris(2-carboxyethyl)phosphine does not have its voltammetric peak within the range of potentials used in the analysis of ascorbic acid, or does not cause distortion of the AA peak. The voltammogram of tris(2-carboxyethyl)phosphine solution with a concentration of 10 mM presented in Figure 2 shows a considerable distortion of the baseline within the range of potentials used in the analysis of ascorbic acid, which indicates the necessity of eliminating it before the voltammetric measurement. For this reason, N-ethylmaleimide was added, as it reacts with phosphine in an acidic environment [24]. The voltammogram obtained as a result of the addition of N-ethylmaleimide to the tris(2-carboxyethyl)phosphine solution, presented in Figure 2, shows a correct baseline within the range of potentials of ascorbic acid. The optimum time necessary for the reagents to complete the reaction was selected as a result of performing a voltammetric analysis of ascorbic acid solution (100 µg/mL) with tris(2-carboxyethyl)phosphine (effective concentration of 10 mM) after the addition of N-ethylmaleimide (effective concentration of 40 mM). A correct result, indicating the elimination of the interference of tris(2-carboxyethyl)phosphine, was obtained after 20 min, and a 30-min time of conducting this reaction was adopted for further analyses (Figure 3).

**Figure 4:**
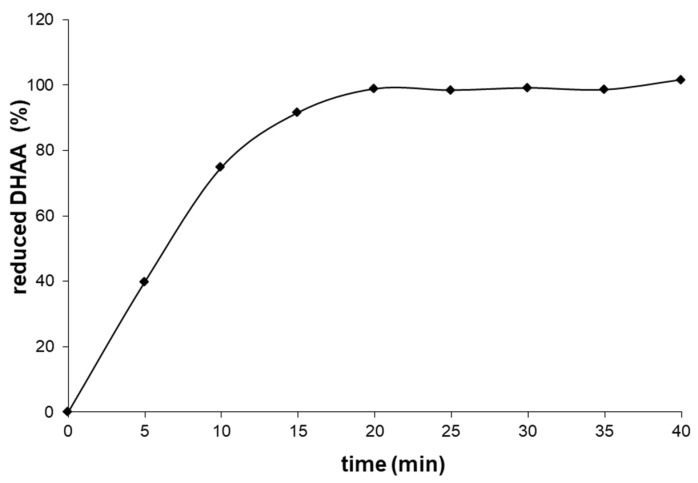
Degree of dehydroascorbic acid reduction versus time with the use of tris(2−carboxyethyl)phosphine.

Research results presented in the literature indicate a possibility of effective reduction of dehydroascorbic acid with the use of tris(2-carboxyethyl)phosphine in the environment of metaphosphoric acid with pH 1.9, as well as hydrochloric acid with pH ≈ 1 [28]. To verify the choice of the concentration of tris(2-carboxyethyl)phosphine solution, the reduction of dehydroascorbic acid with concentration of 1 mM was performed in 2% metaphosphoric acid. Next, 0.2 mL 100 mM of tris(2-carboxyethyl)phosphine solution was added to 1.8 mL of dehydroascorbic acid solution. The solution obtained in this manner was analysed with the HPLC-DAD to determine the time necessary for the achievement of quantitative reduction. Figure 4 presents the relation of the degree of reduction of dehydroascorbic acid in the environment of 2% metaphosphoric acid with time, with the reduction being conducted with the use of tris(2-carboxyethyl)phosphine with effective concentration of 10 mM. The quantitative reduction took place after approximately 20 min. Therefore, it was decided to use a 30-min reduction time in further analyses.

### 2.2. Method Validation

#### 2.2.1. Linearity

**Figure 5:**
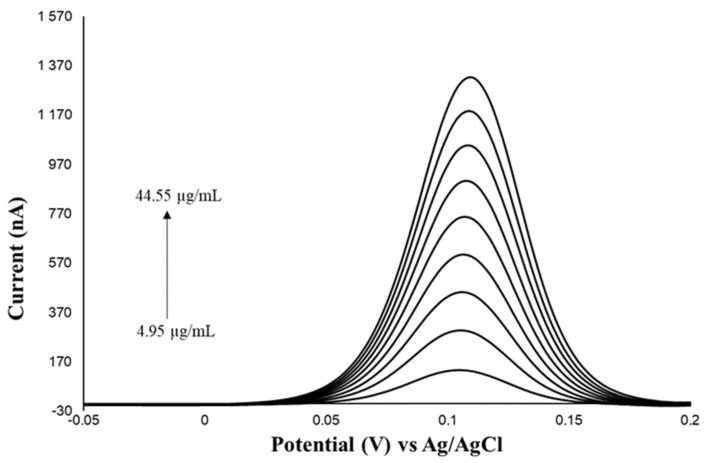
Voltammograms of standard solutions of ascorbic acid plotted for concentrations ranging within 4.95−44.55 µg/mL.

Table 1 presents the results of linear regression and statistical analysis of the determination of standard solutions, the voltammograms of which are presented in Figure 5. As a result of the analysis of the significance of the determined coefficients of the calibration plot, it was found that the value of the slope coefficient differs statistically significantly from zero, and the value of the intercept does not differ statistically significantly from zero, which allows us to conclude about the linearity of the method based on the regression coefficient. Therefore, the determined value of the regression coefficient provides proof of the linearity of the voltammetric method within the range of the analysed concentrations.

#### 2.2.2. Limits of Detection and Quantification

The limit of detection (LOD) was determined based on the parameters of the standard curve obtained on the basis of analysis of standard solutions prepared within the concentration range from 4.95 to 44.55 µg/mL. The limit of detection was calculated on the basis of the value of the standard deviation of the free term s_a_ (4.751 nA) and the residual standard deviation s_xy_ (11.328 nA):

LOD(s_a_) = 0.52 µg/mL

LOD(s_xy_) = 1.25 µg/mL

The inclusion of the residual standard deviation resulted in a higher value of the limit of detection as, in this case, one should also take into account the variation of the slope of the calibration curve. The mean value was adopted as the value of the limit of detection.

LOD = 0.89 µg/mL

The correctness of the determination of the limit of detection was verified [29]. The lowest concentration of the standard solution used was 4.95 µg/mL. Therefore, the conditions indicating the correctness of the determination of the limit of detection are fulfilled:

LOD (0.89 µg/mL) < 4.95 µg/mL

10 LOD (0.89 µg/mL) > 4.95 µg/mL

The limit of quantification (LOQ) was determined on the basis of the formula:

LOQ = 3LOD

LOQ = 2.67 µg/mL

LOQ = 0.53 mg/100 g

The values of the limits of detection and quantification of the voltammetric method and the chromatographic reference method—LOD_HPLC-DAD_ = 0.82 µg/mL, LOQ_HPLC-DAD_ = 2.46 µg/mL [29]—are similar to each other, which indicates the equivalence of the two methods.

#### 2.2.3. Precision

The results of the analysis of food products with the differential pulse voltammetric (DPV) method with the use of tris(2-carboxyethyl)phosphine are presented in Table 2. In the case of analysis of ascorbic acid and of the total content of vitamin C, the coefficient of variation falls within the range from 1.61% to 8.22%, and in the case of dehydroascorbic acid, it is from 4.58% to 66.54%. The analysis of ascorbic acid and the total content of vitamin C yielded Horrat values ranging from 0.33 to 1.29, i.e., values conforming to the recommendations of AOAC [30]. In the case of analysis of dehydroascorbic acid, the Horrat values ranged from 0.5 to 8.02, which indicated imprecise analysis of that acid with the use of the subtraction method, similarly to the use of the chromatographic reference method [29]. The precision of determination of the two methods was compared using the Snedecor F-test. In the case of assays of the content of ascorbic acid and total content of vitamin C for all samples, the analysis of the results of the Snedecor F-test indicates an absence of statistically significant differences of standard deviations. Therefore, the voltammetric method using TCEP is characterized by the same precision as the chromatographic reference method.

#### 2.2.4. Correctness

Analysis of the total content of vitamin C in a sample of the certified reference material BCR 431 and comparison of the obtained average value with the certified value allowed the determination of the correctness of the results obtained. Table 3 presents the obtained data which show an absence of statistically significant differences, indicating the correctness of the results obtained.

Table 4 presents a comparison of the results of the analysis of ascorbic acid, of the total content of vitamin C, and of dehydroascorbic acid, obtained with the voltammetric method with the use of tris(2-carboxyethyl)phosphine, with the results obtained with the chromatographic reference method. The comparison was conducted with the use of the method of calculation of the ratio of mean values (P) and uncertainty (U) of its determination. The results obtained with the two methods are in agreement, but as in the case of the comparison of results obtained with the polarographic method using dithiothreitol, an absence of statistically significant differences in the analysis of dehydroascorbic acid, considerably divergent from one another was noted, which is related with the imprecise analysis [23]. Comparison using Student’s *t*-test indicates an absence of statistically significant differences in the results obtained in the case of all samples, except for the analysis of the total content of vitamin C in tomato and of the content of dehydroascorbic acid in cauliflower.

#### 2.2.5. Recovery

The results of the analysis of the recovery of ascorbic acid in the voltammetric method of vitamin C determination with the use of tris(2-carboxyethyl)phosphine are presented in Table 2. The average values range from 97.66% to 104.20%, and they are in conformance with the requirements of the AOAC about the level of recovery of an analytical method [30]. The results of the Student’s *t*-test indicate an absence of statistically significant differences between the obtained mean value of recovery and the value of 100%.

## 3. Materials and Methods

### 3.1. Materials

The following materials were used: juices from local producers (multi-vegetable, grapefruit, orange), fruits and vegetables (banana, kiwi fruit, cauliflower, broccoli, cucumber, tomato, parsley leaves), fruit cream in powder, infant milk powder, and multivitamin syrup. All samples were purchased from local supermarkets in Lublin, Poland. The certified reference material BCR-431 (Brussels sprout) was purchased from the Institute of Reference Materials and Measurements (IRMM), European Commission Joint Research Centre (Geel, Belgium).

### 3.2. Chemicals

Ascorbic acid, dehydroascorbic acid, N-ethylmaleimide, tris(2-carboxyethyl)phosphine hydrochloride, and mercury standard stock solution (Hg^2+^ = 1 g/L) were purchased from the company Sigma-Aldrich (St. Louis, MO, USA), metaphosphoric acid was purchased from Merck (Darmstadt, Germany), and sodium acetate and glacial acetic acid were purchased from POCH S.A (Gliwice, Poland). All reagents were of analytical grade purity.

### 3.3. Sample Preparation for Analysis

Each sample was weighed to the nearest 1 mg (*m_s_*). Following that, 2% solution of metaphosphoric acid was added and the sample was weighed again (*m_s+a_*). The dilution coefficient (*F*) was calculated and subsequently used to quantify the analyte in the sample.

(1) $$F=\frac{m_{s+a}}{m_{s}}$$

The sample was homogenised for 1 min using an Ultra Turrax (IKA, Staufen, Germany) homogeniser and then centrifuged for 5 min at 12,000× *g*. The supernatant was filtered through a 0.45 µm syringe filter. The obtained extract was divided into two parts; one part was used for the determination of ascorbic acid (AA_p_) and the other was used for the determination of the total content of vitamin C (T_C_) after the reduction of DHAA.

During all stages of analysis, the temperature in the laboratory was between 23 and 25 °C. All analyses were repeated three times, and the results are expressed as mg of analyte/100 g fresh mass in the case of fruits and vegetables and mg of analyte/100 g of product for the remaining samples.

### 3.4. Procedure of Dehydroascorbic Acid Reduction with the Use of Tris(2-carboxyethyl)phosphine

The amount of 0.2 mL 100 mM of tris(2-carboxyethyl)phosphine was added to 1.8 mL of the extract and then the sample was shaken for 30 min on a mechanical shaker. In the case of preparation of a sample for the voltammetric analysis, 2 mL of 80 mM solution of N-ethylmaleimide was added and shaken for 30 min.

### 3.5. HPLC Analysis

The analysis of ascorbic acid was performed using the reversed-phase high-performance liquid chromatography in conformance with the method described by Mazurek and Jamroz [29]. A diode-array detector was used for the detection of analytes. The separation was conducted in the conditions of isocratic elution. In the first step, the content of ascorbic acid in the sample was assayed, followed by the quantitative reduction in dehydroascorbic acid using a reducing agent. Then, the total content of vitamin C was determined. The content of dehydroascorbic acid was calculated by subtracting the initial content of ascorbic acid from the total content of vitamin C. The concentration of ascorbic acid in the extract was determined from the equation of the calibration curve plotted on the basis of the results of the analysis of standard solutions. The identification of ascorbic acid was performed based on the time of retention and the UV spectrum of the reference substance. The analyses were performed with the use of a Varian (Palo Alto, CA, USA) HPLC system equipped with a diode-array detector (DAD, type 335), an isocratic pump (type 210), a dosing valve 7725i (Rheodyne, Cotati, CA, USA), a column thermostat, and a chromatographic column, Gemini 150 × 4.6 mm (3 µm C18), connected with a pre-column Gemini C18 4 × 3 mm (Phenomenex, Torrance, CA, USA). The injection volume was 20 µL. The mobile phase was a solution of orthophosphoric acid at pH 2.8 pumped at the rate of 0.6 mL/min. Chromatograms were recorded at 244 nm and a temperature of 30 °C.

### 3.6. Voltammetric Analysis

Before measurement, the boron-doped diamond electrode (BioLogic Sciences Instruments, Seyssinet-Pariset, France) of 3 mm in diameter was thoroughly polished using a 0.3 μm alumina slurry on a Buehler polishing pad, rinsed with ethanol and water in an ultrasonic bath, and dried with N_2_. The electrode was then transferred into the plating solution containing 20 mg/L Hg(II) and the Hg film was formed by holding the working electrode potential at −1.30 V for 180 s. To minimise the effect of substances present in the matrix of the analysed samples on the electrochemical properties of the electrode, a fresh coat of mercury was applied before the analysis of every sample.

Vitamin C determinations were performed by the method of differential pulse voltammetry, using a voltammetric trace analyser Metrohm 797 VA (Herisau, Switzerland). A three-electrode system was used—a boron-doped diamond electrode modified by the mercury film as the working electrode and a platinum electrode as the auxiliary electrode. The potential of the working electrode was measured in relation to an Ag/AgCl/KCl reference electrode. A quantitative assay was performed with the method of triple standard addition using an automatic dosing unit Dosino 700 (Metrohm AG, Herisau, Switzerland). Qualitative determination of ascorbic acid was performed based on the comparison of the potential of its peak (obtained from sample analysis) with the potential of peaks obtained after adding the standard solution three times to the sample. Ten mL of acetate buffer with pH 4.6 and 0.5 mL of an extract of the analysed sample were added directly to the voltammetric vessel. Before voltammetric measurement, the solution was flushed with argon for 5 min to remove oxygen. The following conditions were applied during the differential pulse voltammetric analysis: initial potential −50 mV, final potential 200 mV, voltage step time 0.6 s, voltage step 6 mV, pulse duration 40 ms, pulse amplitude 50 mV, the rate of potential change 10 mV/s.

The first step consisted of determining ascorbic acid content in the sample (AA_s_ Equation (2)), then quantitative reduction of dehydroascorbic acid was carried out using tris(2-carboxyethyl)phosphine, and total vitamin C content (T_C_) was calculated based on Equation (3). The content of dehydroascorbic acid (DHAA_s_) was calculated by subtracting the initial ascorbic acid content from the total amount of vitamin C (subtraction method).

(2) $$AA_{s}=\frac{C_{AA}\cdot F}{10}$$

where: *C_AA_*—concentration of ascorbic acid in extract (µg/mL)

(3) $$T_{C}=\frac{C_{TA}\cdot 2.22\cdot F}{10}$$

where: *C_TA_*—concentration of ascorbic acid after the reduction step (µg/mL)

### 3.7. Validation

The determination of the linearity, limits of detection and quantification, precision, the accuracy of the new method through the analysis of a sample of certificated reference material, and recovery of the method were performed in conformance with the method described by Mazurek et al. [29]. The comparison of the precision of the methods and the comparison of the results obtained with the use of the chromatographic reference method were performed in conformance with the method described by Mazurek et al. [23].

## 4. Conclusions

This report presents the first description of the development of a new method for the determination of the total content of vitamin C, ascorbic acid, and dehydroascorbic acid in food with the use of the voltammetric technique based on the use of tris(2-carboxyethyl)phosphine as a reducing reagent. The determined validation parameters of the voltammetric method indicate that the method provides correct results of analysis of the total content of vitamin C and ascorbic acid. Analysis of the content of dehydroascorbic acid is imprecise due to the employment of the subtraction method [29]. The validation parameters of the developed method indicate that it is equivalent to the reference method based on the use of high-performance liquid chromatography with spectrophotometric detection.

## Figures and Tables

**Table 1:** The values of parameters calculated on the basis of the calibration curve to determine the linearity of the voltammetric method.

Calibration Curve	y = 29.8957x + 2.5185
Regression coefficient r	0.9996
t_cr_	2.0595
t_b_ *	175.264
t_a_ *	0.5300

* t_cr_—critical value of Student’s *t*-test read from the Tables for significance level α = 0.05 and number of degrees of freedom f = 25. t_b_ and t_a_—parameters of Student’s *t*-test determined for the slope and free term in the linear regression equation.

**Table 2:** Ascorbic acid, total vitamin C and dehydroascorbic acid content in the samples obtained by differential pulse voltammetry and statistical characteristics of the precision of determinations and recovery (t_cr_ = 4.303 and F_cr_ = 19 for α = 0.05).

Sample	Analyte	Mean (mg/100 g)	S * (mg/100 g)	CV * (%)	Horrat *	F *	Recovery ± s (%)	t *
Parsley leaves	AA_s_	204.66	8.01	3.91	0.77	4.04	106.5 ± 15.2	0.73
	T_C_	226.63	4.91	2.17	0.43	2.74		
	DHAA_s_	21.97	12.43	56.57	7.96	3.32		
Tomato	AA_s_	16.87	0.45	2.70	0.36	3.71	98.8 ± 3.8	0.55
	T_C_	17.88	0.55	3.07	0.42	1.06		
	DHAA_s_	1.01	0.11	11.15	0.99	15.74		
Broccoli	AA_s_	76.55	3.62	4.72	0.80	8.80	98.1 ± 8.4	0.38
	T_C_	100.65	4.09	4.07	0.72	3.37		
	DHAA_s_	24.10	2.06	8.54	1.22	2.72		
Cauliflower	AA_s_	91.37	3.61	3.95	0.69	1.84	95.0 ± 12.8	0.67
	T_C_	113.19	5.61	4.95	0.89	3.53		
	DHAA_s_	21.81	2.34	10.74	1.51	3.34		
Banana	AA_s_	11.73	0.78	6.67	0.85	1.72	104.9 ± 5.4	1.56
	T_C_	21.15	0.93	4.39	0.61	1.11		
	DHAA_s_	9.41	1.51	16.09	1.99	1.04		
Lemon	AA_s_	45.04	3.70	8.22	1.29	3.83	103.5 ± 8.4	0.71
	T_C_	53.20	3.35	6.30	1.01	1.04		
	DHAA_s_	7.82	5.20	66.54	8.02	11.17		
Kiwi fruit	AA_s_	108.31	2.85	2.63	0.47	4.85	96.6 ± 10.2	0.57
	T_C_	119.19	2.57	2.16	0.39	7.28		
	DHAA_s_	10.88	1.12	10.27	1.30	2.19		
Cucumber	AA_s_	5.33	0.40	7.45	0.85	1.79	105.4 ± 5.0	1.85
	T_C_	6.16	0.17	2.83	0.33	5.02		
	DHAA_s_	0.83	0.30	36.37	3.13	10.45		
Multivegetable juice	AA_s_	40.79	1.52	3.72	0.58	1.09	95.2 ± 10.5	0.78
	T_C_	42.90	1.02	2.38	0.37	2.65		
	DHAA_s_	2.10	0.89	42.35	4.19	1.02		
Orange juice	AA_s_	21.72	1.17	5.37	0.75	1.80	99.2 ± 9.8	0.14
	T_C_	25.61	1.00	3.92	0.57	2.73		
	DHAA_s_	3.89	0.18	4.58	0.50	11.83		
Grapefruit juice	AA_s_	29.31	0.74	2.51	0.37	2.10	116.3 ± 11.5	2.44
	T_C_	33.34	1.00	2.99	0.45	1.28		
	DHAA_s_	4.03	0.27	6.60	0.72	4.15		
Fruit cream in powder	AA_s_	12.05	0.48	3.98	0.51	1.08	94.6 ± 4.8	1.92
	T_C_	12.54	0.53	4.26	0.55	1.14		
	DHAA_s_	0.49	0.06	12.28	0.98	4.87		
Multivitamin syrup	AA_s_	1043.18	28.93	2.77	0.70	2.12	101.2 ± 8.5	0.23
	T_C_	1132.76	48.18	4.25	1.08	1.47		
	DHAA_s_	89.59	22.44	25.05	4.36	1.41		
Infant milk powder	AA_s_	80.29	2.12	2.64	0.45	1.05	102.4 ± 10.7	0.38
	T_C_	81.97	3.06	3.73	0.64	3.73		
	DHAA_s_	1.68	0.94	56.01	5.35	3.07		
BCR431	AA_s_	468.51	7.55	1.61	0.36			
	T_C_	516.97	14.03	2.71	0.61			
	DHAA_s_	48.46	21.23	43.81	6.94			

* s—standard deviation, CV—coefficient of variation, Horrat—parameter defined as a ratio of the coefficient of variation to coefficient estimated from the Horwitz equation, F—parameter of Snedecor’s F-test, t—parameter of Student’s *t*-test.

**Table 3:** Analysis of the accuracy of the differential pulse voltammetric (DPV) method based on the BCR 431 certified reference material.

BCR 431		DPV				
X_CRM_ * (mg/100 g)	u_CRM_ * (mg/100 g)	X_m_ * (mg/100 g)	u_m_ * (mg/100 g)	Δm *	U_Δ_ *	Accuracy
483	9.8	517	14	34	34.23	yes

* X_CRM_—the certified value, u_CRM_—uncertainty of the certified value, X_m_—the mean value measured, u_m_—the uncertainty of the result of a measurement (expressed as the standard deviation of measurement series), Δm—the absolute difference between the mean measured value and the certified value, U_Δ_—expanded uncertainty of difference between the measured value and the certified value.

**Table 4:** Comparison of ascorbic acid, total vitamin C, and dehydroascorbic acid values obtained by differential pulse voltammetry (DPV) and chromatographic method (HPLC-DAD) (t_cr_ = 2.776 for α = 0.05).

		HPLC-DAD		DPV					
Sample	Analyte	Mean (mg/100 g)	s * (mg/100 g)	Mean (mg/100 g)	s * (mg/100 g)	P *	U *	Accuracy	t
Parsley leaves	AA_s_	201.25	3.98	204.66	8.01	0.983	0.088	yes	0.661
	T_C_	221.91	2.97	226.63	4.91	0.979	0.051	yes	1.422
	DHAA_s_	20.67	6.82	21.97	12.43	0.941	1.330	yes	0.159
Tomato	AA_s_	15.43	0.88	16.87	0.45	0.914	0.122	yes	2.531
	T_C_	16.61	0.53	17.88	0.55	0.929	0.089	yes	2.873
	DHAA_s_	1.18	0.45	1.01	0.11	1.173	0.839	yes	0.657
Broccoli	AA_s_	75.86	1.22	76.55	3.62	0.991	0.100	yes	0.311
	T_C_	97.84	2.23	100.65	4.09	0.972	0.094	yes	1.041
	DHAA_s_	21.98	3.40	24.10	2.06	0.912	0.345	yes	0.923
Cauliflower	AA_s_	88.31	2.66	91.37	3.61	0.966	0.100	yes	1.185
	T_C_	104.35	2.99	113.19	5.61	0.922	0.117	yes	2.410
	DHAA_s_	16.04	1.28	21.81	2.34	0.735	0.282	yes	3.743
Banana	AA_s_	11.53	1.02	11.73	0.78	0.982	0.222	yes	0.278
	T_C_	19.18	0.88	21.15	0.93	0.907	0.127	yes	2.664
	DHAA_s_	7.65	1.49	9.41	1.51	0.813	0.498	yes	1.435
Lemon	AA_s_	48.71	1.89	45.04	3.70	1.081	0.177	yes	1.526
	T_C_	54.03	3.41	53.20	3.35	1.016	0.178	yes	0.303
	DHAA_s_	5.33	1.56	7.82	5.20	0.681	1.653	yes	0.932
Kiwi fruit	AA_s_	106.35	6.27	108.31	2.85	0.982	0.128	yes	0.492
	T_C_	115.31	6.94	119.19	2.57	0.967	0.126	yes	0.907
	DHAA_s_	8.96	1.65	10.88	1.12	0.823	0.402	yes	1.667
Cucumber	AA_s_	4.89	0.30	5.33	0.40	0.916	0.194	yes	1.566
	T_C_	5.88	0.39	6.16	0.17	0.954	0.142	yes	1.149
	DHAA_s_	0.99	0.09	0.83	0.30	1.199	0.693	yes	0.905
Multivegetable juice	AA_s_	38.52	1.46	40.79	1.52	0.944	0.106	yes	1.873
	T_C_	41.09	1.66	42.90	1.02	0.958	0.093	yes	1.601
	DHAA_s_	2.58	0.90	2.10	0.89	1.226	1.082	yes	0.649
Orange juice	AA_s_	20.08	0.87	21.72	1.17	0.925	0.139	yes	1.947
	T_C_	24.36	0.61	25.61	1.00	0.951	0.094	yes	1.840
	DHAA_s_	4.28	0.61	3.89	0.18	1.099	0.313	yes	1.049
Grapefruit juice	AA_s_	30.44	1.07	29.31	0.74	1.039	0.087	yes	1.511
	T_C_	35.20	0.88	33.34	1.00	1.056	0.078	yes	2.419
	DHAA_s_	4.76	0.54	4.03	0.27	1.181	0.275	yes	2.096
Fruit cream in powder	AA_s_	12.18	0.46	12.05	0.48	1.011	0.110	yes	0.332
	T_C_	12.53	0.50	12.54	0.53	0.999	0.117	yes	0.028
	DHAA_s_	0.35	0.13	0.49	0.06	0.717	0.693	yes	1.646
Multivitamin syrup	AA_s_	1053.30	42.09	1043.18	28.93	1.010	0.097	yes	0.343
	T_C_	1096.21	39.70	1132.76	48.18	0.968	0.112	yes	1.014
	DHAA_s_	42.91	18.89	89.59	22.44	0.479	0.885	yes	2.757
Infant milk powder	AA_s_	81.05	2.18	80.29	2.12	1.009	0.075	yes	0.433
	T_C_	83.91	1.58	81.97	3.06	1.024	0.083	yes	0.976
	DHAA_s_	2.86	1.65	1.68	0.94	1.701	1.673	yes	1.075

* s—standard deviation. P—the ratio of means of determination results. U—uncertainty for P value, t—parameter of Student’s *t*-test.

## Data Availability

Not applicable.
